# Executive Function in Young Children: Validation of the Preschool Executive Task Assessment

**DOI:** 10.3390/children12050626

**Published:** 2025-05-13

**Authors:** Yael Fogel, Ortal Cohen Elimelech, Naomi Josman

**Affiliations:** 1 Department of Occupational Therapy, Ariel University, Ariel 40700, Israel; 2 Faculty of Welfare and Health Sciences, University of Haifa, Mount Carmel, Haifa 34980, Israel; ocoheneli@staff.haifa.ac.il (O.C.E.); njosman@univ.haifa.ac.il (N.J.)

**Keywords:** performance-based assessment, ecological validity, cognition, preschool children

## Abstract

Background: Executive function—the cognitive processes and abilities used to perform daily activities and solve real-world problems—is crucial for children’s development. However, existing assessments often lack ecological validity, limiting their ability to reflect real-world cognitive performance. This study aims to validate the Preschool Executive Task Assessment (PETA) as a performance-based ecological measure of executive functions in typically developing Israeli children. Methods: Thirty-six typically developing children participated. Parents completed a demographic questionnaire and the Child Evaluation Checklist, while children undertook two Wechsler Intelligence Scale for Children–Revised 95 subtests. Eligible participants completed three performance-based assessments: PETA, the Children’s Kitchen Task Assessment (CKTA), and the Do-Eat. Inter-rater reliability was examined using the intraclass correlation coefficient (ICC), and concurrent validity was assessed via correlations with the CKTA and Do-Eat scores. The Benjamini–Hochberg correction method was used to control false-positive findings. Results: Age showed weak-to-moderate correlations with key performance measures, including total score, completion time, and required cues (−0.48 < r < −0.37, *p* < 0.05), indicating improved PETA performance with age. Inter-rater reliability for the PETA was high (ICC = 0.84). Significant correlations were found between the PETA completion time and CKTA total score (r = 0.42, *p* = 0.014), and between working memory and the CKTA total score (r = −0.44, *p* = 0.008). Additionally, significant correlations were found between the PETA and the Do-Eat (−0.69 < r < 0.55). Conclusions: Although further research is needed to refine its use across diverse populations and settings with larger samples, these preliminary findings support the PETA’s reliability and validity as a performance-based executive function assessment in young children. This study advances ecologically valid assessments and aids clinicians in selecting appropriate tools for evaluating executive functions in early childhood.

## 1. Introduction

Children rely on executive functions (EFs) to perform everyday tasks, such as schoolwork and social interactions [1]. Executive functions refer to a set of cognitive abilities that emerge in infancy and continue to develop throughout early childhood. These include performance skills such as initiating actions, planning, organizing, sequencing tasks, and self-regulation, all of which are essential for successfully performing self-directed and complicated tasks [2], as well as for regulating their behavior [3]. Given their central role, assessing and supporting EFs in children who may experience deficits—such as those with developmental disorders, including autism spectrum disorder (ASD), attention-deficit/hyperactivity disorder (ADHD), and learning disabilities (LDs)—is essential for the children’s development and integration into society [4,5]. As targeted interventions have the potential to enhance EFs [4], it is important to evaluate EFs early and accurately [6].

Evaluating EFs in this age group presents significant challenges due to the rapid cognitive development occurring during this period [6]. Early childhood is a critical phase in which foundational aspects of EFs, such as inhibitory control, working memory, self-regulation, and mental flexibility, are emerging [2,5,7,8]. As children grow, their ability to perform tasks with higher cognitive demands improves, highlighting the need for an evaluation approach that goes beyond isolated cognitive abilities [8].

However, children’s EFs have traditionally been assessed using neuropsychological assessments focused on specific cognitive components [1]. Although these assessments provide valuable insights into distinct EFs, they often lack ecological validity, meaning they do not accurately reflect real-life performance [9]. Everyday tasks, such as participating in social play or engaging in arts and crafts, typically require the simultaneous use of multiple EFs, making traditional neuropsychological assessments less representative of daily cognitive demands [9].

Performance-based assessments offer greater ecological validity by providing a more accurate depiction of EFs in real-world settings [1]. Ecological performance-based tasks that involve multiple steps are especially useful for translating assessment findings into practical applications, such as in classroom environments [10,11]. However, the extent to which performance on these assessments generalizes to real-world contexts and activities beyond those included in the assessment tool remains unclear. This underscores the need for further research into the validity and applicability of performance-based assessments [2]. Furthermore, there are limited valid tools for detecting EF deficits in young children [12].

The Preschool Executive Task Assessment (PETA) [6], adapted from the Children’s Kitchen Task Assessment (CKTA) [1], is specifically designed to assess EFs in young children. In the PETA, children complete a self-directed educational activity involving multiple steps. They assemble a caterpillar picture using provided materials while following a visually guided instruction book. This dynamic assessment incorporates a cueing system that highlights the child’s strengths, weaknesses, and overall performance, offering a more comprehensive evaluation of their EFs [6].

Unlike traditional assessments that emphasize accuracy, the PETA focuses on the process of task completion. This focus allows a more ecologically valid evaluation of EFs in a way that reflects children’s real-world performance, particularly in classroom settings. The PETA is coded both qualitatively and quantitatively and is not time-restricted. It provides insights into various aspects of EFs, including the time required for task completion, the ability to initiate the task independently, transitions between steps, distractibility, and the level of support needed to complete the task successfully [6].

The PETA’s reliability and validity have been investigated in previous research. The test was administered to 166 typically developing children aged 3 to 5 years living in London. In 10 of these sessions, video recordings were independently scored by three raters blinded to participant details. Inter-rater reliability (ICC) was 0.93, indicating strong agreement. Furthermore, PETA performance significantly improved with age, as younger children required more support across all quantitative EFs domains, with girls requiring less support compared to boys. Concurrent validity was established through comparisons with the Behavior Rating Inventory of Executive Function–Preschool Version (BRIEF-P), showing that children who required more cues in the PETA also exhibited poorer EFs as reported by their parents [6]. The PETA has been translated into Turkish [13,14] and has been used with children with sickle cell anemia [6].

However, research has not yet compared the PETA with other ecological performance-based assessments. Evaluating its standardization in comparison to similar tools is essential to ensuring its accuracy and real-world applicability. Moreover, cultural and environmental factors can influence children’s executive function development and performance on structured assessments [15]. The PETA has not been evaluated among Israeli children, making it crucial to assess its suitability for this population.

Examining the PETA among typically developing Israeli children can provide valuable insights into its usability and potential for use in diverse educational and clinical settings. Therefore, this study aims to achieve the following:

1. Determine the relationships between gender and age and the PETA assessments;
2. Establish the PETA’s inter-rater reliability;
3. Establish the PETA’s concurrent validity with the CKTA [1] and Do-Eat [15] assessments.

## 2. Materials and Methods

### 2.1. Participants

The study sample consisted of 36 typically developing children aged 5 to 8 years (M = 6.79, SD = 0.88). Participants were recruited through community advertisements targeting typically developing young children. Information about this study was disseminated through social media platforms (e.g., Facebook) and relevant online groups (e.g., WhatsApp groups). Inclusion criteria required participants to achieve an average score of 10 (SD = 3) on the vocabulary and block design subtests of the Wechsler Intelligence Scale for Children–Revised 95 (WISC-R95). Additionally, participants had a mean score of 3.61 (SD = 0.26) on the Child Evaluation Checklist (CHECK) [16]. Exclusion criteria included any diagnosed psychiatric, emotional, or autism spectrum disorders, physical disabilities, or neurological conditions as reported by parents.

### 2.2. Procedure

The Ethics Committee of the Faculty of Health Sciences, Ariel University, approved this study on 1 March 2022 (AU-HEA-YF-20200507). Before participating, all parents provided written informed consent. This study involved one 90-min session, including breaks, attended by parents and children. During the session, parents completed the demographic questionnaire and the CHECK while the children undertook two WISC-R95 subtests. Following an evaluation of their eligibility for this study, the children completed three assessments: the Preschool Kitchen Task Assessment (PKTA), the CKTA, and the Do-Eat. The assessments were administered in a counterbalanced order across participants to mitigate order effects and potential biases.

### 2.3. Instruments

#### 2.3.1. Wechsler Intelligence Scale for Children–Revised 95

The WISC-R95 Hebrew version [17] assesses verbal and nonverbal capacities to evaluate intellectual functioning. Only its vocabulary and block design subtests were administered to access these capabilities, define participants’ profiles, and rule out participants with possible intellectual disabilities. The mean score of each subtest (vocabulary and block design) is 10 with a range of three standard deviations. Sattler [18] reported that the combination of the vocabulary and block design subsets has a strong correlation with the WISC-IV full-scale IQ (r = 0.92) and adequate test–retest reliability (r = 0.87). These tests are commonly used to assess intellectual function in evaluation studies in the Israeli population of children with complex neurodevelopmental disabilities [19,20,21].

#### 2.3.2. Child Evaluation Checklist

Functional information was collected from the CHECK [16], which helps identify developmental delays based on parental reports. This tool includes two parts. The CHECK-A reports the child’s current functioning level, with a focus on frequency, using 30 statements rated on a Likert scale from 1 (never) to 4 (always). Example statements include, “Completes tasks he or she takes upon him- or herself,” and “Correctly estimates difficulty of the task”. The CHECK-B also rates the participant’s functioning but focuses on a comparison to expectations for same-aged peers. It uses 10 statements rated from 1 (low) to 5 (high). Example items include, “Compared to other children, the overall functioning of my child is …”, and “In the area of work habits, the overall functioning of my child is …”.

An average score is calculated for each part. Internal consistency had a Cronbach’s alpha (α) of 0.96 for the CHECK-A and 0.94 for the CHECK-B. Construct validity was established and documented in previous articles [3]. The structure validity [16] was examined compared to the BRIEF-P [22], the Beery–Buktenica Developmental Test of Visual Motor Integration [23], and the Star-Wave Test [24]. In the current study, we used only the CHECK-A with α = 0.93.

#### 2.3.3. Preschool Executive Task Assessment

The PETA was adapted from the CKTA [1]. This assessment examines the EFs of preschool-aged children by determining the level of assistance they require to complete a specific task—assembling a picture of a caterpillar using a box of required materials. At the beginning of the task, the assessor provides the participant with an instruction book with pictures of the steps needed to assemble the caterpillar craft. In addition, the assessor tells the child that all necessary materials are in the box provided and that the child should try to complete the task independently.

The PETA scores are determined by the level of assistance (cues) needed to complete each step of the task: 0 (no cues), 1 (verbal guidance), 2 (gesture guidance), 3 (direct verbal assistance), 4 (physical assistance), and 5 (do for participant). Participants receive cues after 10 s to allow for processing and problem-solving. However, if they appear to be in an unsafe situation or at risk of damaging the arts-and-crafts project, the researcher provides the cues earlier. The scoring system consists of two components: (1) the total number of cues given throughout the task, and (2) a weighted score ranging from 1 to 5 that accounts for the relative impact of each type of cue. At the completion of the task, each participant’s level of assistance, time, total cue count, and weighted score are calculated to provide a comprehensive measure of performance.

The PETA includes a before-task questionnaire, which the examiner administers to participants prior to the assessment. The child is asked to indicate how much assistance they believe will need during the task: none, a little, or a lot. After completing the task, the child is asked again to reflect on the actual level of assistance they require. Additionally, they are asked to evaluate their performance using a four-point scale: excellent, good, not so good, or poor. The assessment was translated into Hebrew and then back-translated with the authors’ approval to ensure accuracy and consistency.

#### 2.3.4. Children’s Kitchen Task Assessment

The CKTA [1] is a performance-based measure of executive functioning. Children follow a word-and-picture recipe to make play dough, receiving structured cues as needed. The cueing sequence progresses from general verbal guidance to physical assistance, with a score of 0 indicating no assistance required. Each cue level is given twice before advancing to the next.

Inter-rater reliability was established with 22 subjects, yielding a high intraclass correlation coefficient (ICC = 0.98). Internal consistency was moderate (α = 0.68), likely reflecting the combination of five EF abilities. Working memory is embedded in tasks requiring the repeated use of utensils and timers.

The CKTA assesses EF categories including initiation, planning/sequencing, judgment/safety, and completion. Scoring includes time to completion, total cues given, a weighted score (cues × cue level), organization score, and individual EF domain scores. Scores range from 0 to 400, with higher scores indicating a greater need for cues and lower EF abilities. The assessment was translated into Hebrew with the authors’ approval.

#### 2.3.5. Do-Eat Assessment

The Do-Eat assessment is a standardized, performance-based tool for children aged 5 to 8 years, conducted in natural settings [15]. It consists of three tasks: making a sandwich, preparing chocolate milk, and completing a certificate of achievement. The evaluator introduces and demonstrates each task before the child performs it.

Performance is assessed across three dimensions: task performance, sensorimotor skills, and EFs. The EFs include attention, initiation, sequencing, transitions, spatial and temporal organization, inhibition, problem-solving, and memory. Children receive cues when needed, with scores ranging from 5 (independent) to 1 (requiring physical assistance).

Parents complete a 10-item questionnaire evaluating their child’s EFs and daily task performance at home. The final Do-Eat score is based on the total of task performance, sensorimotor, and EF scores, while cueing and parental questionnaire scores guide intervention planning.

This assessment demonstrates strong psychometric properties, with good internal consistency (task performance = 0.73, sensorimotor skills = 0.87, EFs = 0.87, parental questionnaire = 0.89), good inter-rater reliability (0.92), and construct validity [9,15,25].

### 2.4. Data Analyses

All statistical analyses were conducted using IBM SPSS (vers. 29), with a significance threshold set at *p* < 0.05. Descriptive statistics were used to summarize demographic and performance data. Correlation analyses examined associations between demographic variables (e.g., age, gender) and PETA scores. Inter-rater reliability was calculated using ICCs to determine the level of agreement between raters. Concurrent validity was assessed by analyzing correlations between the PETA, CKTA, and Do-Eat performance scores. To control false-positive findings due to multiple comparisons, we applied the Benjamini–Hochberg correction method [26]. We set the false discovery rate (FDR) at 10%, using a significance threshold of *p* = 0.05 [27].

## 3. Results

### 3.1. Sample Demographics

Table 1 summarizes the demographic characteristics and the findings of the measures related to the inclusion criteria. In general, the sample consisted of 36 typically developing children, of which more than half were boys. Ages ranged from 5.1 years to 8.5 years. All children except one were enrolled in regular education programs, and all were Hebrew speakers. Most of their parents had higher education degrees (fathers *n* = 32, 89%; mothers *n* = 35, 97%).

### 3.2. Gender and Age Relationships with PETA

**Figure 1:**
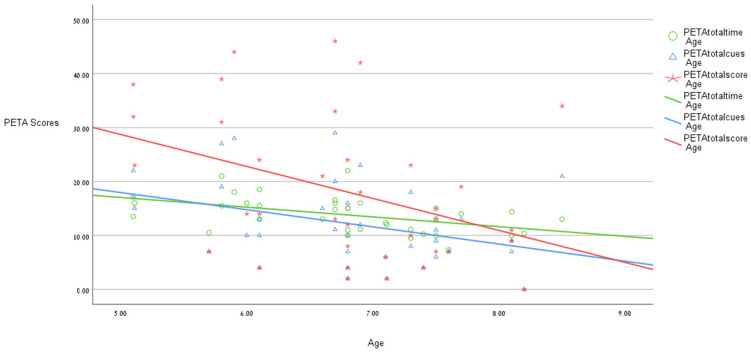
The correlation between gender, age, and PETA quantitative measures.

**Figure 2:**
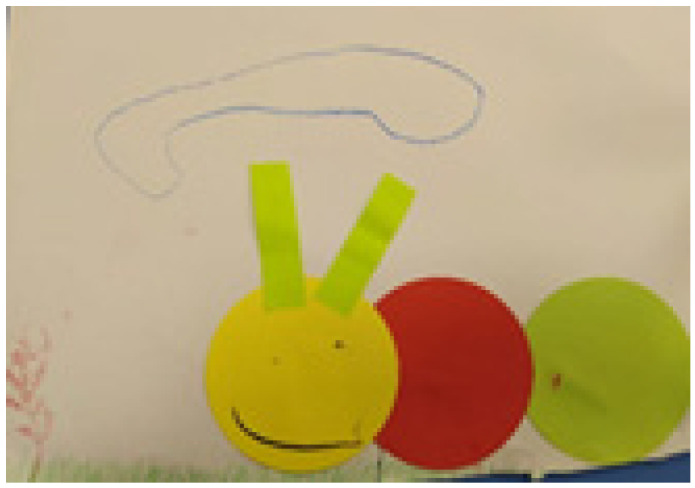
Example PETA outcome, younger child.

**Figure 3:**
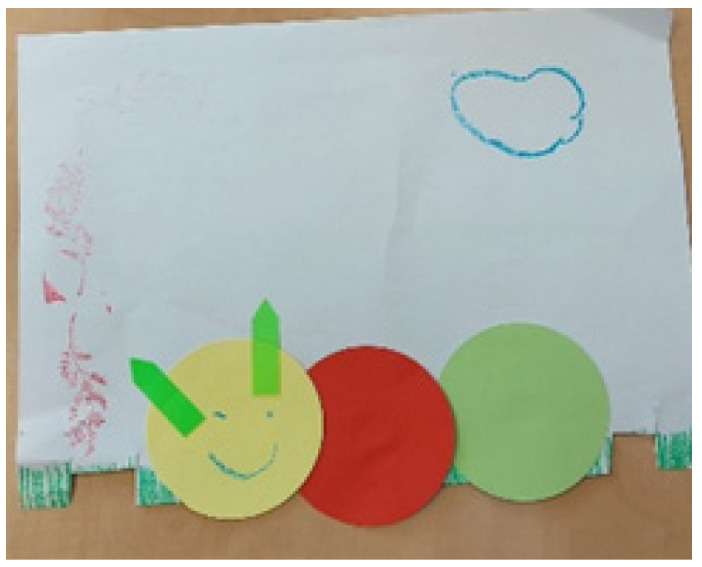
Example PETA outcome, older child.

Except for gender and age, the correlation analysis between the demographic variables and the PETA scores revealed no significant correlations. Gender revealed a significant correlation only with the highest level of support required during the task (r = −0.44, *p* < 0.05), and age had weak-to-moderate correlations with qualitative scores, including distractibility and emotional control (respectively, r = 0.52, *p* = 0.01 and r = 0.35, *p* < 0.05). Additionally, age showed weak-to-moderate correlations with performance on certain quantitative measures, such as the total score, completion time, and total number of cues required (−0.48 < r < −0.37, *p* < 0.05). Figure 1 illustrates the findings, showing that as age increased, performance on the PETA improved. Specifically, with increasing age, the PETA total completion time, number of cues required, and total score decreased, indicating better performance. Figure 2 and Figure 3 provide examples of the PETA outcomes of two children, one younger than the other.

### 3.3. Reliability Analysis

#### 3.3.1. Inter-Rater Reliability

Inter-rater reliability for the PETA was assessed with two evaluators and four children using the ICC to measure agreement between independent raters. The analysis yielded an ICC of 0.84, indicating a high level of agreement. This finding supports the inter-rater reliability of the PETA, ensuring consistency in scoring across different evaluators.

#### 3.3.2. Concurrent Validity

Table 2 and Table 3 present the significant correlations between the final scores of the PETA and the CKTA and between the PETA and the Do-Eat, respectively. The adjusted *p*-values, calculated using the Benjamini–Hochberg procedure, are presented. Results demonstrate a significant moderate correlation between the PETA completion time and CKTA total score (r = 0.42, *p* = 0.014). Additionally, a moderate significant negative correlation was found between working memory and the CKTA total score (r = −0.44, *p* = 0.008), indicating that higher working memory capacity is associated with better performance on the CKTA. However, no significant correlations were found for PETA total cues, organization, emotional liability, or distractibility.

Regarding the correlations between the PETA and the Do-Eat, significant weak-to-moderate correlations (0.47 < r < 0.71) were noted between the PETA and the Do-Eat. However, no significant correlations were found between the PETA’s completion time, organization, and distractibility scores and any of the Do-Eat variables—total score, total cue score, qualitative variables, or the Do-Eat’s total time score.

## 4. Discussion

In this study, we established the reliability and validity of the PETA among a group of Israeli children, a dynamic assessment designed to evaluate EFs. The findings suggest that the PETA demonstrates strong inter-rater reliability and aligns with established EF measures, supporting its construct (concurrent) validity. Additionally, this study identifies significant age-related correlations with EF performance, potentially providing insights into developmental trajectories and their implications. Nevertheless, the results are preliminary and should be interpreted with caution due to the sample size and the exploratory nature of this study.

### 4.1. Gender- and Age-Related Correlations with EF Performance

Regarding gender, no significant correlations were found, suggesting that PETA performance is similar for both genders in this age group. Yet, further research with a larger sample size is needed to confirm these findings.

As expected, age was significantly correlated with PETA performance. Younger children required more cues and longer completion times and demonstrated greater distractibility and emotional variability. These findings align with research indicating that EF skills develop progressively during early childhood, with improvements in working memory, self-regulation, and inhibitory control [4,7]. The observed relationship between age and EF measures, in combination with previous research reporting similar findings, reinforces the importance of developmental benchmarks in the interpretation of PETA scores.

### 4.2. Inter-Rater Reliability

The PETA demonstrated strong inter-rater reliability (ICC = 0.84), indicating a high level of agreement among independent raters. This result aligns with previous research on the CKTA, which yielded similar reliability measures (ICC = 0.93) [1]. High inter-rater reliability ensures the PETA provides consistent and objective assessments across evaluators, reinforcing its potential for clinical and educational applications. In addition, it enhances the validity of this study’s findings [28].

### 4.3. Concurrent Validity

Significant correlations were found between the PETA scores and both the CKTA and Do-Eat assessments, supporting the concurrent validity of PETA as an EF measure. Concurrent validity (a part of criteria validity) with the CKTA showed significant moderate correlations for completion time and working memory Given that the PETA was developed based on the CKTA, this correlation is expected; both assessments evaluate overlap in EFs, such as task sequencing, problem-solving, and cognitive flexibility. However, no significant correlations were found for PETA total cues, total score, organization, emotional lability, or distractibility, possibly indicating that the PETA may be more sensitive in capturing qualitative aspects of EFs than the CKTA. Further research is needed to explore these correlations in a larger and more representative sample.

The significant correlations between the PETA and Do-Eat, which captures broader performance abilities scores, suggest that the PETA measures similar cognitive components, particularly those related to task initiation, planning, and execution [15,25]. The PETA’s intuitive design—especially its environmental conditions and material requirements—and its significant correlations with the well-established Do-Eat assessment [25] reinforce its criteria validity in assessing cognitive functions and highlight its potential clinical utility.

However, no significant correlation was found between the PETA’s completion time, organization, and distractibility scores and any of the Do-Eat variables. This result possibly reflects differences in task structure and the measured types of EFs each assessment tool emphasizes. Specifically, the Do-Eat assesses sensorimotor and fine motor coordination skills alongside EFs, whereas the PETA focuses on cueing levels and self-directed performance.

### 4.4. Limitations and Future Directions

Despite its contributions, this study has several limitations. First, the sample size was relatively small (*N* = 36), which may limit the generalizability of the findings. Future research should use larger and more representative samples to enhance statistical power. Additionally, validation studies should be expanded to include clinical populations (e.g., children with ADHD, ASD, or learning disabilities and with lower cognitive abilities) to assess the PETA’s diagnostic utility and discriminant validity. Furthermore, the PETA’s convergent validity should be examined by comparing it with other EF measures, such as parent-reported EF ratings (e.g., the BRIEF-P).

Second, although the PETA has been translated into Hebrew, cultural differences in educational practices and social expectations may influence EF performance [15,29]. Comparative studies across cultural settings would be valuable in determining the assessment’s cross-cultural applicability.

Finally, it is important to acknowledge that the PETA, CKTA, and Do-Eat involve varying types of tasks, each requiring distinct levels of cognitive demand. Future research should investigate how task complexity and structure affect EF performance. Furthermore, because EFs develop over time, a longitudinal study design would provide deeper insights into changes in PETA scores with age and intervention effects. These insights can enhance the PETA’s construct validity, particularly its ability to discriminate between age groups.

## 5. Conclusions

This study supports evidence for the reliability and validity of PETA as a performance-based EF assessment for young children. Its high inter-rater reliability and significant correlations with existing tools support its potential for real-world applications. Although further research is needed to refine its use across diverse populations and settings with larger samples, the PETA offers a valuable contribution to EF assessment. It bridges the gap between traditional neuropsychological tests and ecologically valid performance-based evaluations. Thus, the findings may encourage the adoption of a broader perspective, as represented in the concept of functional cognition. Functional cognition refers to a child’s ability to carry out daily activities by integrating metacognitive processes, executive functions, motor skills, and established habits and routines. Instead of focusing on specific cognitive abilities, this approach emphasizes individuals’ ability to complete activities in real-life contexts [10].

From a clinical perspective, incorporating the PETA, CKTA, and Do-Eat into the clinician’s toolbox enhances their ability to assess EFs using different methods, each with distinct strengths. The CKTA and PETA rely on visual instructions presented to the child, guiding them through structured tasks. They also offer accessible assessment options that are easier to administer in various clinical and educational settings because they do not require a fully equipped kitchen or additional materials. In contrast, the Do-Eat requires the child to initiate the activity independently, simulating a more naturalistic task, such as preparing chocolate milk. This study’s findings contribute to the development of ecologically valid assessments and support clinicians in selecting the most appropriate tool.

## Figures and Tables

**Table 1:** Demographic characteristics and inclusion criteria findings.

Characteristic	*n* (%)
Gender	
Male	22 (61%)
Female	14 (39%)
Social-communal family status	
Low	1 (3%)
Average	13 (36%)
High	22 (59%)
	M (SD) Range
WISC-R95	
Vocabulary	9.61 (2.94) 6–17
Block design	11.42 (2.86) 3–17
CHECK	3.47 (0.38) 2.83–4.23

Note. *N* = 36.

**Table 2:** Correlations between the final scores of the Preschool Executive Task Assessment (PETA) and the Children’s Kitchen Task Assessment (CKTA).

PETA Variable	CKTA Variable		
	Completion Time	Total Score	Total Number of Cues
	r (p)		
Quantitative variable			
Completion time		0.42 (0.014) ^a^	
Total score			
Total cues			
Qualitative variable			
Working memory		−0.44 (0.008) ^a^	
Organization			
Emotional lability			
Distractibility			
	M (SD)		
	24.73 (4.96)	26.37 (15.91)	18.01 (8.96)

^a^ The *p*-values after applying the Benjamini–Hochberg correction.

**Table 3:** Correlations between PETA final scores and Do-Eat scores.

PETA Variable		Do-Eat					
		Total Time	Total Score	Final Cue Score	Sensory Motor Skills	Executive Function Skill	Task Performance
	M (SD)	r (p)					
Quantitative variable							
Completion time	13.82 (3.33)						
Total score	13.32 (18.14)		−0.69 (<0.001) ^a^		−0.59 (<0.001) ^a^	−0.64 (<0.001) ^a^	−0.68 (<0.001) ^a^
Total cues	12.28 (7.62)		−0.71 (<0.001) ^a^		−0.61 (<0.001) ^a^	−0.64 (<0.001) ^a^	−0.69 (<0.001) ^a^
Qualitative variable							
Working memory	2.17 (0.56)		0.53 (<0.001) ^a^			0.47 (0.004) ^a^	0.55 (<0.001) ^a^
Organization	2.33 (0.53)						
Emotional lability	2.44 (0.61)					0.51 (0.002) ^a^	
Distractibility	2.22 (0.63)						
		M (SD)					
		32.42 (6.78)	13.27 (0.98)	11.84 (2.83)	4.61 (0.33)	4.35 (0.44)	4.29 (0.38)

^a^ The *p*-values after applying the Benjamini–Hochberg correction.

## Data Availability

The datasets generated and/or analyzed during the current study are not publicly available due to ethical restrictions but are available from the corresponding author on reasonable request.

## Abbreviations

The following abbreviations are used in this manuscript:

ADHD	Attention-deficit/hyperactivity disorder
ASD	Autism spectrum disorder
BRIEF-P	Behavior Rating Inventory of Executive Function–Preschool Version
CHECK	Child Evaluation Checklist
CKTA	Children’s Kitchen Task Assessment
EFs	Executive functions
ICC	Interclass correlation coefficient
PETA	Preschool Kitchen Task Assessment
WISC-R95	Wechsler Intelligence Scale for Children–Revised 95
